# Developing European guidelines for training care professionals in mental health promotion

**DOI:** 10.1186/1471-2458-12-1114

**Published:** 2012-12-27

**Authors:** Tim Greacen, Emmanuelle Jouet, Peter Ryan, Zoltan Cserhati, Vera Grebenc, Chris Griffiths, Bettina Hansen, Eithne Leahy, Ksenija Maravic da Silva, Amra Šabić, Angela De Marco, Paz Flores

**Affiliations:** 1 Etablissement public de santé Maison Blanche, Paris, France; 2 Middlesex University, London, United Kingdom; 3 Semmelweis University, Institute of Behavioural Sciences, Budapest, Hungary; 4 Faculty of Social Work, University of Ljubljana, Ljubljana, Slovenia; 5 Human Resources Department, Capacity Building Unit, Central Region, Denmark; 6 Hospital del Mar Medical Research Institute, Barcelona, Spain; 7 University of Primorska, Koper, Slovenia; 8 University of Molise, Campobasso, Italy; 9 Centre Fòrum, Institut de Neuropsiquiatria i Adiccions, Hospital del Mar Medical Research Institute, Barcelona, Spain

**Keywords:** Mental health promotion, Training, Best practice guidelines

## Abstract

**Background:**

Although mental health promotion is a priority mental health action area for all European countries, high level training resources and high quality skills acquisition in mental health promotion are still relatively rare. The aim of the current paper is to present the results of the DG SANCO-funded PROMISE project concerning the development of European guidelines for training social and health care professionals in mental health promotion.

**Methods:**

The PROMISE project brought together a multidisciplinary scientific committee from eight European sites representing a variety of institutions including universities, mental health service providers and public health organisations. The committee used thematic content analysis to filter and analyse European and international policy documents, scientific literature reviews on mental health promotion and existing mental health promotion programmes with regard to identifying quality criteria for training care professionals on this subject. The resulting PROMISE Guidelines quality criteria were then subjected to an iterative feedback procedure with local steering groups and training professionals at all sites with the aim of developing resource kits and evaluation tools for using the PROMISE Guidelines. Scientific committees also collected information from European, national and local stakeholder groups and professional organisations on existing training programmes, policies and projects.

**Results:**

The process identified ten quality criteria for training care professionals in mental health promotion: embracing the principle of positive mental health; empowering community stakeholders; adopting an interdisciplinary and intersectoral approach; including people with mental health problems; advocating; consulting the knowledge base; adapting interventions to local contexts; identifying and evaluating risks; using the media; evaluating training, implementation processes and outcomes. The iterative feedback process produced resource kits and evaluation checklists linked with each of these quality criteria in all PROMISE languages.

**Conclusions:**

The development of generic guidelines based on key quality criteria for training health and social care professionals in mental health promotion should contribute in a significant way to implementing policy in this important area.

## Context

In the 2008 *European Pact for Mental Health and Well-being*[1], the European Commission encourages Member States to engage in longer-term cooperation on mental health and well-being in the European Union. It recognises the health, social and economic benefits of good mental health for all and the need to overcome the taboo and stigma still associated with mental illness. To achieve this, the Pact recommends, as a priority area for action for European States, ‘*promot(ing) training of professionals involved in the health, education, youth and other relevant sectors in mental health and well-being’* (p.4) [1]. Promo-ting mental health has to be seen as a long-term investment requiring long-term education and information programmes. Similarly, on a more international level, the World Health Organisation (WHO)’s 2004 report on the *Prevention of mental disorders*[2] underlines the importance of capacity building and training health professionals in the area of mental health promotion, urging programme implementers to provide guidelines for training and supervision. Capacity building and training are key issue not only in terms of programme implementation, but also for policy-making, research and advocacy. The report recommends international collaborations to promote training initiatives. The question of which actors should receive training is subject to debate. For the WHO’s *Mental Health Action Plan for Europe,* the current priority for mental health in Europe is, above all, to train primary healthcare professionals [3]. In terms of mental health promotion, this would imply not just integrating appropriately trained mental health professionals into general health or social care services but, more importantly, integrating mental health promotion skills into other professionals’ everyday practice and skills repertoires. For lower income countries however, integrating mental health into primary care may not be the best solution: using low-cost approaches such as reinforcing traditional social support around young mothers in the months following childbirth or relying on community health workers drawn from the communities they serve, may be preferable [4].

Training health and social care professionals in mental health promotion is all the more important in that the principal systematic reviews on this question have found many interventions to be of varying degrees of effectiveness [5,6]. This is so despite the existence of a wide range of evidence-based programmes and policies available for implementation [7], and of specific best practice instruments for translating the health promotion evidence base into practice [8] and helping health promotion specialists improve the effectiveness of their interventions [9]. The recent European Psychiatric Association’s guidance paper on mental health promotion [10] asserts the importance of embracing the principle of mental health promotion, but makes little reference to training psychiatrists or other health professionals in this area. Currently, the workforce for mental health promotion varies considerably from one country to the next. Although countries such as the Netherlands have created specialised “mental health promotion and prevention workers”, high level training resources and quality skills acquisition are rare in Europe, as is training for the quality implementation of mental health promotion programmes. In most countries, undergraduate and postgraduate education in mental health promotion is not a separate specialisation [11]. Various training programmes do however exist, including training manuals for health care professionals in primary care to work with families, training in planning and evaluation in low income countries, training aimed at changing the organisation of health services to promote a more holistic approach to health, and training for parents to promote child mental health [7,12]. The 2003–2008 IMHPA (Integrating Mental Health Promotion Interventions into Countries’ Policies, Practice and Mental Health Care System) project included representatives of 14 European Union countries, five countries in accession (at the time) and Norway, representatives of four European networks and the World Health Organization. The project developed an internet database of evidence-based mental health promotion and mental disorder prevention programmes [13], as well as a training manual for primary health care professionals on mental health promotion for adults based on developing problem-solving skills [14]. The 2004–2006 MINDFUL (Mental health information and determinants for the European Level) project devised a training programme in mental health promotion and prevention interventions as one of its major project outcomes [15]. The 2007–2010 PROMO (Best Practice in Promoting Mental Health in Socially Marginalized People in Europe) project interviewed experts in 14 European countries to identify four components of best practice in promoting mental health in socially marginalised people: establi-shing outreach programmes; facilitating access to services that provide different aspects of health care including mental health care; strengthening the collaboration and co-ordination between different services; and disseminating information both to marginalised groups themselves and to professionals [16]. Training does not only concern health and social actors. The MINDFUL project focused on setting up a comprehensive system for European mental health monitoring purposes, including a set of 35 mental health indicators [17]. This project led to the development of a three day training course for health professionals and programme implementers, but also for experts and researchers in national institutes or in non-governmental organisations engaged in mental health promotion, aimed at building capacity in terms of use of mental health indicators across European member states.

Within this context, the present article describes how the PROMISE project developed generic guidelines for training social and health care professionals in mental health promotion in Europe.

## Method

### Scientific committee and partners

The study was part of the PROMISE project (*Promoting Mental Health, Minimising Mental Illness and Integrating Social Inclusion through Education*), which was funded by the European Commission (DG SANCO) and conducted from 2009 to 2012 [18]. The PROMISE project was carried out at eight sites in seven countries: Aarhus, Denmark; Paris, France; Budapest, Hungary; Molise, Italy; Koper and Ljubljana, Slovenia; Barcelona, Spain; and London, the United Kingdom. The project brought together a multidisciplinary scientific committee representing a variety of institutions including three universities providing mental health training programmes, four mental health service providers with continuous training remits and a public health organisation. The scientific committee was made up of site leaders and researchers from each site. All scientific committee members had experience in the area of either (a) mental health promotion and prevention in social or health care, or (b) training social or mental health care professionals. Professions represented in the scientific committee included clinical and research psychology, pedagogic research, social work, general medical practice and psychiatry. Mental health service users and their organisations participated in each phase of the project.

### Document analysis

The methodological approach was adapted to the two major specificities of the scientific committee. Firstly, although most partner countries had national or local policy on mental health promotion, there was little identifiable existing expertise on training professionals on this subject. Secondly, a crucial aspect of the present project involved bringing together in the scientific committee and in local steering groups, stakeholder and professional groups from different sectors who were not accustomed to working together, for whom the same terms did not necessarily mean the same things and who did not possess the same knowledge, competency or experience base. Standard techniques for identifying best practices, such as for example the Delphi process, which tend to iron out potentially powerful but discordant voices too early on in the process, were not considered appropriate. We therefore chose to use a participative stakeholder empowerment approach in three phases. In the first phase, all scientific committee members participated in a two-day training session to become acquainted with each other and acquire the standard thematic analysis research techniques necessary for analysing the scientific and policy literature. The second phase involved an iterative feedback strategy with local steering groups at each site, examining the literature in question and agreeing upon themes that, together, they felt to be important. This year-long process allowed participants across the eight sites and from all different stakeholder and professional groups to develop (a) a common knowledge base on mental health promotion training and policy in this area, and (b) best practice themes identified and accepted by all partners in all countries. During the third phase, the resulting guidelines then underwent a second feedback process, in which local steering groups tested the guidelines and their associated resource kits during local mental health training programmes, targeting different care professions and different health promotion themes. Feedback from each site was progressively submitted to monthly internet meetings of the scientific committee and integrated into the PROMISE Guidelines and their associated resource kits.

In the initial step, all site leaders and all members of the scientific committee participated in a two-day training programme on mental health promotion and thematic content analysis techniques [19,20] with the aim of building a Document Analysis Template, an initial coding frame to be used for the analysis of the principal EU policy documents and EU-funded scientific literature and policy reviews on mental health promotion to identify information concerning training professionals on this subject. During this face-to-face meeting, in order to guarantee that each scientific committee member mastered the necessary research techniques for the analysis that was to follow, codes were developed by all members of the scientific committee collectively reading the European Pact for Mental Health and Well-Being [1] line by line, based on the principles of thematic content analysis. Each member was asked to identify texts within the document that they considered in any way to be linked to the principal research question of good practice in training professionals in mental health promotion. Recurring themes were identified, and each theme was given a brief definition and illustrated with examples. For example, the need to adopt an interdisciplinary and intersectoral approach in mental health promotion was rapidly identified as a recurring theme. An illustration of this comes from the European Pact itself, which underlines, with regard to mental health and well-being in young people, the importance of promoting training of professionals in the health, education, youth and other relevant sectors [1].

The resulting coding frame was then used to code the two major existing EU-funded literature and policy reviews on mental health promotion in Europe: the IMHPA Project [21] and the MINDFUL Project [22]. Discrepancies in coding between centres were identified and clarified during online scientific committee meetings across all sites. In cases where new codes were identified, these were discussed and included into the coding frame.

The new Data Analysis Template was then used to analyse eight further policy documents and scientific literature reviews. Saturation was reached rapidly: although all documents were found to mention training, few explored quality criteria in any detail. At the end of this process, three further documents concerning specific themes that had been mentioned but not developed in the first 11 documents were added for analysis. These documents concerned: mental health policy in low and middle-income countries [23], mental health promotion for mental health service users [24], and community capacity building [25].

### Documents analysed by PROMISE Scientific Committee members

The following 14 documents were analysed by all scientific committee members at all PROMISE sites:

1. Slovenian Presidency of the European Union: *European Pact for Mental Health and Well-being*. Brussels; 2008 [1].http://ec.europa.eu/health/ph_determinants/life_style/mental/docs/pact_en.pdf

2. Jané-Llopis E, Anderson P: *Mental Health Promotion and Mental Disorder Prevention: a policy for Europe*. Nijmegen: Radboud University Nijmegen; 2005 [21].http://ec.europa.eu/health/archive/ph_projects/2002/promotion/fp_promotion_2002_a01_16_en.pdf

3. STAKES, MINDFUL - Mental health information and determinants for the European level (2006) [22]http://info.stakes.fi/NR/rdonlyres/A8D81F03-CD9C-463A-9374-E5251BA9E841/0/Consolidatedfinalreport.pdf

4. National Institute for Health and Clinical Excellence: *Public health interventions to promote positive mental health and prevent mental health disorders among adults. Evidence briefing*. London; 2007 [26].http://www.nice.org.uk/niceMedia/pdf/mental%20health%20EB%20FINAL%2018.01.07.pdf

5. Queen Mary University of London: *Best Practice In Promoting Mental Health In Socially Marginalized People In Europe*. London; 2008 [27]. http://www.promostudy.org/index.html

6. Jané-Llopis, E. & Anderson, P. (Eds): *Mental health promotion and mental disorder prevention across European Member States: a* collection *of country stories.* Luxembourg: European Communities; 2006 [11]http://www.gencat.cat/salut/imhpa/Du32/html/en/dir1662/dd11714/country_stories.pdf

7. Wahlbeck K. & Mäkinen M. (Eds): Prevention of depression and suicide. Consensus paper. Luxembourg: European Communities; 2008 [28]. http://ec.europa.eu/health/ph_determinants/life_style/mental/docs/consensus_depression_en.pdf

8. World Health Organization Department of Mental Health and Substance Abuse: *Prevention of Mental Disorders: effective interventions and policy options*. Geneva; 2004. [3]http://www.who.int/mental_health/evidence/en/prevention_of_mental_disorders_sr.pdf

9. World Health Organization Department of Mental Health and Substance Abuse: *Promoting mental health: concepts, emerging evidence, practice: summary report.* Geneva; 2004 [7]. http://www.who.int/mental_health/evidence/en/promoting_mhh.pdf

10. Jané-Llopis E, Barry M, Hosman C, Patel V: The evidence of mental health promotion effectiveness: strategies for action. Promotion and Education 2005 (Suppl 2). [12]http://www.gencat.cat/salut/imhpa/Du32/html/en/dir1663/Dd12975/iuhpe_special_edition_no2.pdf

11. World Health Organization Regional Office for Europe: *Mental health action plan for Europe: facing the challenges, building solutions. Report from the WHO Ministerial Conference.* Copenhagen; 2005 [3]http://www.euro.who.int/__data/assets/pdf_file/0008/96452/E87301.pdf

12. Patel V, Araya R, Chatterjee S, Chisholm D, Cohen A, De Silva M, Hosman C, McGuire H, Rojas G, van Ommeren M: **Treatment and prevention of mental disorders in low-income and middle-income countries.***Lancet* 2007, **370**:991–1005 [23]. http://www.ncbi.nlm.nih.gov/pubmed/17804058

13. Pape B, Galipeault J-P: Mental Health Promotion For People With Mental Illness - A Discussion Paper, Public Health Agency of Canada: Ottawa; 2002 [24]. http://www.phac-aspc.gc.ca/publicat/mh-sm/mhp02-psm02/pdf/mh_paper_02_e.pdf

14. Austen P: *Community Capacity Building and Mobilization in Youth Mental Health Promotion: the Story of the Community of West Carleton. How the Community Helper Program Evolved from a Community’s Experience with Youth Suicide*. Ottawa: Health Canada Health Promotion Unit; 2003 [25]. http://www.phac-aspc.gc.ca/mh-sm/mhp-psm/pub/community-communautaires/pdf/comm-cap-build-mobil-youth.pdf

### Iterative feedback process with local steering groups

The next stage of the analysis consisted in merging the 50 identified codes into categories and their subsequent refinement and grouping into main conceptual themes [29,30]. The resulting quality criteria were then presented to local steering groups at each site. Each local steering group was required to include, in addition to the members of the local PROMISE research team: mental health professionals with experience in mental health promotion, service user stakeholders, and training professionals. Feedback from local steering groups was integrated into the Guidelines using a consistent methodology across the collaborating PROMISE sites, to arrive at findings that are not limited to specific groups or specific national contexts. In this manner, the final ten themes were confirmed as the ten PROMISE Guidelines quality criteria for training professionals in mental health promotion.

### Resource kit development process

At this point, the Guidelines were then subjected to a second iterative feedback procedure with the local steering groups. The aim of this second feedback procedure was to develop, for each of the ten quality criteria, resource kits and evaluation checklists in all participating languages, to be used when creating generic mental health promotion training programmes, or programmes for specific care professions or on specific themes. Steering groups also gathered information from national or local policy texts, stakeholder groups and professional organisations on existing local training programmes or policies. Details of this process of evaluating the PROMISE Guidelines across the eight European sites are described elsewhere, including testing the Guidelines with regard to particular care professions [31,32] and specific health issues [33,34]. Feedback from each site was progressively submitted to monthly internet meetings of the scientific committee and integrated into the Guidelines.

## Results

The PROMISE Scientific Committee identified ten final quality criteria for training health and social care professionals in mental health promotion:

### The Ten PROMISE Quality Criteria for Training Professionals in Mental Health Promotion

Quality training programmes for health and social care professionals on mental health promotion should respect the following ten quality criteria:

1. ***Embracing the Principles of Mental Health Promotion:*** The training programme embraces the idea of mental health promotion as distinct from mental illness prevention or curative care. Positive mental health is seen as a resource, as a value on its own and as a basic human right essential to social and economic development. Mental health promotion aims to impact on determinants of mental health so as to increase positive mental health and to reduce inequalities. [http://promise-mental-health.com/qualitycriteria/qc1.html?v=1]

2. ***Empowering All Community Stakeholders for Effective Involvement:*** The training programme embraces the principle of community participation and involvement. Mental health promotion involves encouraging and empowering all community stakeholders in mental health promotion in general or in developing specific mental health promotion projects. In the case of training professionals for specific mental health projects, representatives from the populations directly concerned by the mental health promotion objective in question are encouraged to participate in fixing the health objectives and designing and delivering the programme. The training programme also takes into account how the populations concerned are going to be able to resource and manage their health promotion in a sustainable way (finance, time, etc.). [http://promise-mental-health.com/qualitycriteria/qc2.html?v=1]

3. ***Adopting an Interdisciplinary and Intersectoral Approach:*** The training programme takes into account the necessarily interdisciplinary and intersectoral approach to mental health promotion. It aims for all stakeholders to have collective ownership of the training programme and of the mental health promotion interventions associated with the programme. It encourages the acquisition of leadership skills to build shared vision, shared planning and strategy for mental health promotion actions. [http://promise-mental-health.com/qualitycriteria/qc3.html?v=1]

4. ***Including People with Mental Health Problems:*** The training programme applies its objectives also to people with experience of mental health problems, mental health service users and their carers. People with mental health problems and, in the case of training related to a particular mental health promotion programme, people with mental health problems related to the programme objective, are included from the outset. [http://promise-mental-health.com/qualitycriteria/qc4.html?v=1]

5. ***Advocating:*** The training programme underlines the importance of advocacy, i.e. knowing how to bring out and defend the point of view of people who may not have the skills or the social power necessary to defend themselves or, in the policy arena, working for positive change in the social or health care system environment. [http://promise-mental-health.com/qualitycriteria/qc5.html?v=1]

6. ***Consulting the Knowledge Base:*** The training programme takes into account up-to-date scientific evidence and ethnographic information, drawing from a variety of methods, including epidemiology and social sciences, for identifying action strategies. [http://promise-mental-health.com/qualitycriteria/qc6.html?v=1]

7. ***Adapting Interventions to Local Contexts and Needs in a Holistic, Ecological Approach:*** The training program highlights the fact that interventions to promote mental health must be adapted to local contexts and needs (taking into account the context in which people live) and to the individuals involved. From the individual’s perspective, this means treating specific mental health objectives in a holistic manner, taking into account the particularities of the community and the physical environment the individual lives in, and taking into account different cultures, socio-economic and educational situations, age, gender, sexual orientation, health and abilities. The training program is built around health promotion objectives that are measurable and can be evaluated; and the communities or individuals in question are involved in this process of evaluation: assessing local needs, choosing objectives and indicators and evaluating results. [http://promise-mental-health.com/qualitycriteria/qc7.html?v=1]

8. ***Identifying and Evaluating Risks:*** The training programme addresses not only the expected positive outcomes but also the possible risks of the mental health promotion intervention(s) being presented for both individuals and communities. All changes involve a degree of risk-taking. Empower individuals and communities to decide the level of risk they are prepared to take with their health and safety. [http://promise-mental-health.com/qualitycriteria/qc8.html?v=1]

9. ***Using the Media:*** The training programme integrates a media and communication strategy in promoting mental health and fighting against stigma associated with mental illness. [http://promise-mental-health.com/qualitycriteria/qc9.html?v=1]

10. ***Evaluating Training, Implementation and Outcomes: ***The training underlines the importance of monitoring implementation processes as well as evaluating mental health promotion training and programme outcomes in general. [http://promise-mental-health.com/qualitycriteria/qc10.html?v=1]

The complete PROMISE European Guidelines for Training Professionals in Mental Health Promotion, including resource kits and checklists in seven EU languages, as well as applications to the specific training needs of psychiatrists, psychologists, nurses and social workers, can be consulted at: http://promise-mental-health.com[35].

### Feedback from local steering groups

The results from the second iterative feedback procedure with local steering groups underlined the importance of developing comprehensive resource kits attached to each of the ten quality criteria. For many local stakeholders, the concept of mental health promotion itself was new. Others pointed out that a closer look at existing local mental health promotion programmes often revealed that the programmes actually being implemented were simply traditional prevention programmes. Local steering groups underlined the importance of giving examples of mental health promotion programmes with the different populations that training participants may be working with. Social and health care professionals stressed the fact that most qualified care professionals have only received training in preventive or curative approaches to mental health. It was therefore considered essential that the resource kits be adapted not only for undergraduate “initial” training on mental health promotion in general but also for postgraduate or continuous training on specific mental health promotion themes or projects. Carer and user members of the steering groups insisted that, if they were to participate in training care professionals, the language used in the guidelines should be kept as simple as possible, with a glossary of terms for participants not coming from academia or who do not have the same professional or experiential background. Similarly, resource kits should include material and references in their local European languages, to facilitate participation of stakeholder groups who were not necessarily fully familiar with the English language. Finally, references and links were systematically and comprehensively made with sister European project, CompHP (Developing Competencies and Professional Standards for Health Promotion Capacity Building in Europe) [36], which has produced recommendations on core competencies for general health promotion. Similarly, links were made with the work of the European Network for Mental Health Promotion [37] which is developing mental health promotion handbooks and e-learning programmes with a particular focus on schools, workplaces and older people’s residences [38].

The final PROMISE Guidelines resource kits contain, for each of the ten quality criteria, a detailed description of the quality criterion in question, definitions of the principle terms used, key policy documents related to that criterion, examples of existing training programmes and mental health promotion interventions that respect this quality criterion, and a checklist for quality evaluation, as well as links to posters, brochures, relevant internet resources, and stakeholder websites. All this is available in the languages of all members of the PROMISE consortium [35]. For example, the resource kit attached to the first quality criterion (QC1) defines the key terms and concepts used, paying particular attention to using terminology that remains accessible to all stakeholders. Concepts such as the ‘right to health’, ‘health inequalities’ or ‘health determinants’ are presented as follows: *“Health promotion is not just about changing attitudes and behaviours, but also about defending people’s right to health and changing living conditions. Inequalities in society can lead to poorer mental health for those with less mental health resources. Mental health promotion involves acting upon the determinants of mental health, including clients’ and communities’ social and ecological conditions, income, employment, housing, leisure, daily routines, transport, social and physical environment.”* The same resource kit then goes on to present a list of international training programmes that embrace the notion of mental health promotion as distinct from but not excluding mental illness prevention. Each programme is briefly described. Programmes address issues such as: social and emotional well-being; resilience; interpersonal skills; the cultural diversity of notions of well-being; mental health as a right; understanding loss and grief; using and abusing drugs and alcohol; developing parenting skills. Feedback from local steering groups also revealed the importance of the checklists associated with each quality criterion. Checklist items such as, for QC1, “*The training programme underlines how to impact on the determinants of mental health so as to increase positive mental health and to reduce inequalities”* or “*The training programme refers to promoting mental health in general or with regard to a specific mental health theme, and not just to preventing mental illness”* proved particularly useful in helping training designers evaluate how well their programmes respected the quality criterion in question.

## Discussion

The PROMISE Project research process identified ten final quality criteria around which were then constructed the PROMISE Guidelines for training social and health care professionals in mental health promotion. The first of these criteria (QC1) underlines the fact that training programmes in this area need to *embrace the principles of mental health promotion* as distinct from mental illness prevention or curative care. Positive mental health is to be seen as a resource, as a value on its own and as a basic human right essential to social and economic development. Mental health promotion seeks to act on the determinants of mental health in order to promote positive mental health and optimise the psychological development of the whole population, including those who are suffering from mental health problems or who are seen to be at risk. A mental health promotion approach thus includes mental illness prevention as one of its expected outcomes, but refuses to equate mental health with the simple absence of illness [2].

The following three criteria identify different actors that need to be involved in mental health promotion. Firstly, *empowering all community stakeholders for effective involvement* (QC2) is essential if each is to play a meaningful and sustained role in the programmes being implemented. Secondly, *adopting an interdisciplinary and intersectoral approach* (QC3) is at the centre of all contemporary mental health promotion capacity building policy. The 2005 WHO European Declaration on Mental Health famously asserts that: *“There is no health without mental health. Mental health is central to the human, social and economic capital of nations and should therefore be considered as an integral and essential part of other public policy areas such as human rights, social care, education and employment”*[3]. This cannot be achieved by the health sector alone. Indeed, effective mental health promotion involves acting on the social, ecological and economic determinants of health: enhancing social connectedness and social inclusion, fighting against discrimination and violence, adapting the physical environment, living conditions, transport and schools. Given the diversity and multiplicity of the determinants of human well-being in any given situation, mental health promotion is intrinsically intersectoral. It should involve a broad range of professionals not only from the health, education, social and justice sectors, but also other stakeholders such as civil society organisations, and user and carer organisations [3,7,21]. Multidisciplinary, intersectoral teamwork should be included in the training of all actors. This is particularly true for mental health staff in the context of deinstitutionalisation, with carers and other community stakeholders, including primary care professionals, playing an increasing role in providing support for people with mental health problems [3].

An essential component of this training is the development of a common language between specialists from different professional backgrounds working in different sectors, aiming for common core competencies and capacities such as skills in communication, management, facilitating implementation and evaluation in order to strengthen working partnerships in mental health promotion projects [3]. Finally, QC4, *Including people with mental health problems*, underlines the fact that mental health service users and carers have legitimate roles to play in participating in developing and conducting training for professionals in mental health promotion and also in promoting mental health in general. One of the major reasons why mental health promotion programmes fail concerns the stigma in most European societies with regard to mental health issues. Involving people with mental health problems in the design and delivery of these programmes has been shown to be a key way (a) to combat this stigma and (b) to design messages and actions that avoid creating new reasons for stigmatisation [11]. Service users’ experiences of living with mental illness and of the stigma associated with mental illness, need to be utilised in a proactive way in the design and delivery of mental health promotion programmes. Building user skills for participation in training and advocacy is therefore a priority in mental health promotion [24]. Thus training professionals in mental health promotion necessarily includes a process of inclusion of service user trainers in these programmes.

Capacity building and training are also key issues for effective *advocating* (QC5) and for *consulting the knowledge base* (QC6), both of which are identified as specific chapters in the final PROMISE guidelines. Building health promotion advocacy networks based on up-to-date scientific evidence and ethnographic information, drawing from a variety of methods, including epidemiology and social sciences for identifying action strategies are key ways for bringing different sectors to work together effectively. Understanding advocacy skills is particularly important when defending and integrating the point of view of socially excluded groups, such as Roma populations, undocumented migrants or the long-term unemployed. Introducing advocacy skills into front-line services, with the aim of developing effective mental health promotion programmes, requires specific training of both health and social care staff, as well as interpreters [16].

A weakness of many capacity building programmes in the area of mental health promotion is to neglect questions of training and support with regard to programme implementation. The quality of programme delivery, the skill and style of delivery and the way the programme integrates participants’ and stakeholders’ different points of view, will strongly influence participant responsiveness and engagement [36]. Furthermore, research on this issue is scanty: little is known about the impact of training on knowledge, attitudes and behaviour with regard to mental health promotion [39]. Three PROMISE guideline criteria address these questions. *Adapting interventions to local contexts and needs in a holistic, ecological approach* (QC7) and *identifying and evaluating risks* (QC8) are key to promoting programme delivery skills that will empower individuals and communities to decide the level of risk they are prepared to take with their health. This is particularly true with regard to addressing stigma about mental illness and people with mental illness, a major barrier to accessing not only care, but also information on mental health in general. Training professionals in *using the media* (QC9), including the Internet and online social networks, has proven to be effective in challenging stigma, increasing peer support and promoting positive attitudes towards mental health issues in general [11]. In the same vein, the European Parliament Resolution of 19 February 2009 on Mental Health calls for the development of European guidelines for responsible coverage of mental health by the media.

Finally, quality training for health and social care professionals in *evaluating training, implementation and outcomes* (QC10) is key to success. Mental health promotion also involves acquiring skills with regard to evaluating not only the training itself, but also the implementation and outcomes of the mental health promotion objectives being targeted in the training. QC10, which asserts the principle of evaluation, also has implications for respecting criteria such as QC7 which insists that the different stakeholders in the communities involved in a given mental health promotion programme should also be involved in evaluating that programme.

There were a number of limitations to the study. In the methodology, the categorisation process inevitably simplified the data, reducing differences of interpretation and the richness of local experiences. The resource kits attached to each quality criterion do to some extent recreate the richness of cultural diversity between sites. However, these are in seven different languages, and remain relatively inaccessible to partners not fluent in these languages. Only the checklists, the EU-funded documents and the English language documents were accessible to all PROMISE partners. Further limitations include the fact that the members of the scientific committee, the local steering groups and local experts were chosen on the basis of local knowledge or research team membership: recruitment was therefore to some extent opportunistic and may have been inconsistent. Finally, the selection of documents to be analysed, consisting almost entirely of EU-funded policy documents and literature reviews, and in spite of continuing to examine further documents exploring specific themes well after saturation had been reached, will inevitably influence external validity. Generalisation to settings outside European contexts should take this into account.

On the other hand, the study does have a number of strengths. A substantial number of professionals and stakeholder groups were consulted across eight sites in seven European countries. The scientific committee was composed not only of mental health professionals, but included representatives from social work, predagogic research and general medical practice, as well as mental health service users. Similarly, partners included a variety of institutional categories including universities providing mental health training programmes, mental health service providers with continuous training remits and a public health organisation. All scientific committee members participated in all steps of developing the document analysis template, coding, identifying themes and the resulting ten quality criteria, and integrating feedback from consultation with partners at local sites. The final list of ten guideline criteria reflects commonalities across countries and research teams despite national, institutional or professional differences, and therefore may be seen to be widely applicable.

## Conclusions

In conclusion, the final PROMISE Guidelines for training health and social care professionals in mental health promotion are built around ten quality criteria, each with its associated resource kit and checklist. These Guidelines are the result of a three year process based on a thematic analysis of policy documents and scientific literature reviews, and integrating feedback from local and European professional and stakeholder groups across eight European sites. It is hoped that the PROMISE Guidelines will contribute in a significant way to implementing policy in this important area.

## Competing interests

The authors declare that they have no competing interests.

## Authors’ contributions

TG, EJ, PR, ZC, VG, CG, BH, EL, KM, AS, AD and PF all made substantial contributions to the design of the study, data collection, interpretation of the findings and critical revision of drafts. All authors read and approved the final manuscript.

## Pre-publication history

The pre-publication history for this paper can be accessed here:

http://www.biomedcentral.com/1471-2458/12/1114/prepub
